# Rhabdomyosarcoma of the head and neck: 24 cases and literature review

**DOI:** 10.1590/S1808-86942010000400020

**Published:** 2015-10-19

**Authors:** Giovana Moretti, Ricardo Guimarães, Karisa Martins de Oliveira, Fernanda Sanjar, Richard Louis Voegels

**Affiliations:** a Otorhinolaryngologist, fellowship in endonasal endoscopic surgery, otorhinolaryngological unit, Clinical Hospital, Medical School, São Paulo University (HCFMUSP); b Otorhinolaryngologist, fellowship in endonasal endoscopic surgery, otorhinolaryngological unit, HCFMUSP; c Pediatric oncologist at the Child Cancer Therapy Insttute, Itaci/Child Insttute, ICR-HCFMUSP; d Otorhinolaryngologist at the otorhinolaryngological unit, HCFMUSP; e Assistant professor in otorhinolaryngologist at the otorhinolaryngological unit, HCFMUSP

**Keywords:** head and neck, neoplasms, soft tissue neoplasms, rhabdomyosarcoma

## Abstract

Rhabdomyosarcoma (RMS) is a malignant tumor of soft tissues, more common in childhood, mainly located in the head and neck. It presents varied clinical and biological behavior and requires individualized management.

**Aim:**

To describe information on patients with head and neck RMS diagnosed and treated in a hospital, and to compare them to results in the literature. Study design: Descriptive and retrospective.

**Materials and Methods:**

A retrospective analysis of data from 24 patients with head and neck rhabdomyosarcoma diagnosed and treated in a hospital from 1994 to 2008.

**Results:**

The mean age was 7.79 years. According to gender, 54.17 % were males and 45.83 % were female. All patients underwent chemotherapy (CT), 62.5% of them also underwent radiotherapy (RT) and 16.67% were submitted to surgery. Of the 24 patients, 8 (33.3%) died, 6 (25%) were found free of neoplasia and 2 (8.3%) experienced tumor recurrence.

**Conclusion:**

The RMS of the head and neck often presents with nonspecific symptoms. Individualized multimodal therapy should be performed for these patients, including surgery, chemotherapy and radiotherapy.

## INTRODUCTION

Sarcomas are rare mesenchymal malignant neoplasms comprising less than 1% of diagnosed cancers in the US and about 1% of head and neck cancers in adults.^1^ Over 50% of head and neck malignant tumors in children are soft tissue sarcomas and lymphomas.

Salivary gland carcinomas, nasopharyngeal carcinomas, neuroblastomas, and thyroid carcinomas are other less common head and neck tumors in children.^2^

The rhabdomyosarcoma, a skeletal muscle subtype, is the most common soft tissue sarcoma in children,^3^ comprising about 50% of these tumors.^4^ In contrast, adult rhabdomyosarcomas comprise less than 10% of all soft tissue sarcomas. Its incidence is 3.5% in children aged 0 to 14 years, and 2% in teenagers aged 15 to 19 years.^5^

The head and neck are the most common primary sites for rhabdomyosarcomas in children and teenagers, followed by the genitourinary tract, limbs, thorax, and retroperitoneum.^3^^,^^5^

The tumor head and neck subsites include the orbit, parameningeal sites (nasopharynx, nasal cavity, paranasal sinuses, temporal bone, pterygopalatine fossa, and the infratemporal fossa), and non-parameningeal sites.^6^^,^^7^ Tumors that invade the orbit only have a better prognosis.^8^

Subtypes may be histologically classified as: embryonic (which may be subdivided into embryonic, botryoid, and spindle cell tumors), alveolar, or pleomorphic.^3^^,^^5^^,^^7^^,^^8^ In children, about 60% are embryonic tumors, 20% are alveolar tumors, 15% are not classified, and 5% are pleomorphic tumors.^5^ The embryonic subtype has a better prognosis in children, but is more aggressive in adults. The alveolar subtype has a poor outcome because of its propensity to metastasize at a distance. The pleomorphic subtype occurs predominantly in adults.^4^^,^^9^^,^^10^ The majority of rhabdomyosarcoma cases occurs randomly, with no risk factors, although a small proportion of these tumors are associated with genetic conditions.^5^

The diagnosis should include a complete medical history, a physical examination, a complete blood count, the blood chemistry profile including liver enzymes, nasal endoscopy, computed tomography (CT), magnetic resonance imaging (MRI) and a biopsy for pathology.^3^ Signs and symptoms depend on tumor location. Sometimes the tumor may not be detected in the physical examination, but may become apparent because of pain or functional disorders.^11^ The tumor often presents as a painless neck mass.^2^ Nasal block, rhinorrhea and recurring otitis media are the most common presenting symptoms.^9^ Rapidly progressive eye proptosis is a common manifestation in tumors of the orbit.^11^ There are no pathognomonic endoscopic findings in rhabdomyosarcomas. Some tumors in the nasal cavity - or altered mucosa - and small tumors in the paranasal sinuses where the sinus walls remain intact may not be detected endoscopically.^9^^,^^12^

CT shows the extent of disease and its relation with vital structures. Bone remodeling suggests benign or slow-growing tumors, while bone destruction and loss of soft tissue suggest malignancies. MRI provides superior soft tissue resolution; fat suppression around the facial sinuses, the pterygopalatine fossa and the infratemporal fossa increase the sensitivity of this method for tumor extension. MRI also differentiates the tumor, muscles, secretions, and mucous thickening better, and is superior when assessing perineural and perivascular structures, and intracranial invasion.^2^^,^^12^

The prognosis of patients with rhabdomyosarcomas depends on the primary site, tumor size, the histological subtype and tumor staging, the latter according to the Intergroup Rhabdomyosarcoma Study Group (IRSG) criteria, which is based on disease extent, and local and regional resectability (Table 1).^5^^,^^9^ Tumors of the orbit have a better prognosis; parameningeal (including nasal cavity tumors), nasopharyngeal, paranasal sinus, middle ear, mastoid, infratemporal fossa, and pterygopalatine fossa tumors have a worse prognosis because of the possibility of subarachnoid spread.^2^^,^^3^^,^^4^^,^^8^

**Table 1:** Groups for surgery and pathology^5^

GROUP	DEFINITION
I	Localized tumor, fully resected with free surgical margins and no regional lymph node involvement
II	Localized tumor, resected macroscopically: (A) microscopic disease along the margins, (B) affected regional lymph nodes, (C) both A and B
III	Localized tumor, with residual disease after incomplete resection or biopsy only
IV	Distance metastases present upon the diagnosis

Having established a diagnosis of rhabdomyosarcoma, the next step is to evaluate the extent of disease before initiating therapy.

The clinical assessment includes a thoracic CT, a bone marrow biopsy, a bone scintigraphy, and a cranial base and brain MRI.^5^ The lungs are the most common site for distance metastases.

Treatment should be individualized for each patient.^8^ Survival in patients with head and neck rhabdomyosarcomas has increased with multidisciplinary therapy, which comprises chemotherapy, surgery, and radiotherapy.^2^^,^^6^^,^^7^

Patients with recurring rhabdomyosarcomas have a worse prognosis. The choice of therapy is based on several factors such as the site of recurrence or prior therapy.^5^

The purpose of this study was to review 24 cases of patients that were diagnosed and treated in a specific hospital and to compare the data with findings in the literature.

## SERIES AND METHOD

A retrospective review was undertaken of the records of 24 patients with head and neck rhabdomyosarcomas that were diagnosed and treated at a specific hospital from 1994 to 2008.

A descriptive analysis was made of age, sex, the clinical presentation of tumors, the histological subtype, the site of the primary tumor, and the type of therapy; these patients were monitored and the data was compared with findings in the literature, as reviewed in Medline.

## RESULTS

**Chart 1:**
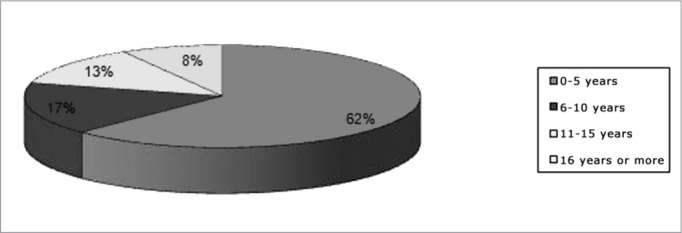
Distribution of patients according to age.

There were 24 patients, 13 (54.17%) male and 11 (45.83%) female. The mean age was 7.79 years (standard deviation − 9.51). The earliest diagnosis was made at birth, and the most advanced age at diagnosis was 47 years (Chart 1).

**Chart 2:**
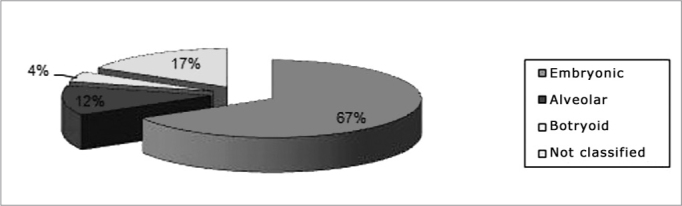
Distribution according to the histological subtype.

The distribution according to the histological types was as follows: 16 embryonic (66.67%), 3 alveolar (12.5%), and 1 botryoid (4.17%); the subtype was not classified in 4 patients (16.67%) (Chart 2).

The interval between the onset of symptoms and the final diagnosis was about two months in most patients.

**Chart 3:**
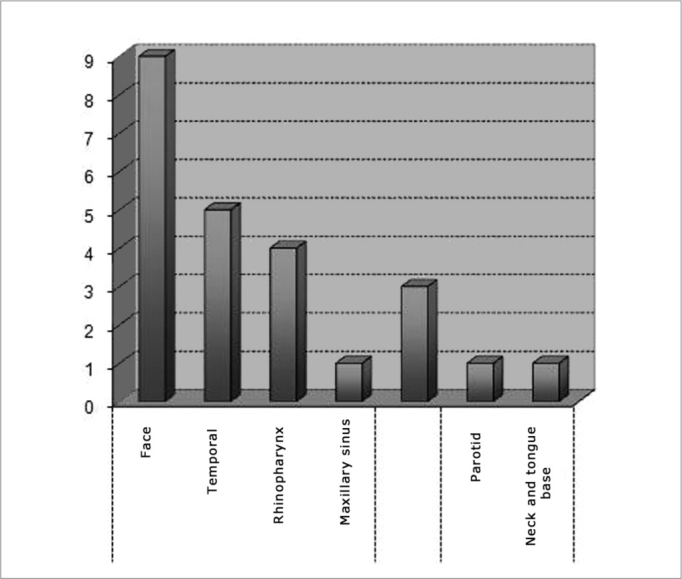
Distribution according to the subsite.

Tumor subsites were parameningeal in 19 cases (79.1%), orbitary in three cases (12.5%), and parameningeal in two cases (8.3%). Among the parameningeal tumors the primary sites were: 9 cases in the face (47.3%), 5 cases in the temporal region (26.3%), 4 cases in the rhinopharynx (21.05%), 1 in the maxillary sinus (5.2%). Of the parameningeal tumors, one was located in the parotid and one in the neck and base of tongue (Chart 3).

The clinical picture and the physical examination at diagnosis and after varied depending on the tumor site. Patients with orbitary subsites course with rapidly progressive bulging and inflammation in the orbit. Peripheral facial otalgia and paralysis were commonly found in patients with mastoid rhabdomyosarcomas. Symptoms and physical findings varied when the primary tumor was located in the face and rhinopharynx; these included bulging, facial asymmetry or cellulitis, nasal block, rhinorrhea, snoring, epistaxis, odynophagia, facial paralysis, and loss of weight. A few patients were initially mistakenly treated for sinusitis, otitis or tonsillitis.

All patients underwent CT and MRI of the face and cranium to assess the tumor site, extent, and intracranial invasion. Intracranial invasion was found in 10 patients (41.67%); metastases at the time of diagnosis or during the progression of disease were present in 12 patients (50%). The main metastatic sites were the bones, lungs, meninges, the central nervous system, and neck lymph nodes.

Chemotherapy was done in all patients. Supplementary radiotherapy was done in 15 patients (62.5%); only 4 patients (16.67%) underwent surgery, which consisted of partial resection in two cases and orbit exenteration in two cases. Death ensued in 8 patients; 6 patients were tumor-free at the time of discharge, and were followed up in the outpatient unit. Recurrence occurred in two patients one year after the end of therapy. Other patients were lost to follow-up and could not be found to provide information.

## DISCUSSION

The rhabdomyosarcoma is the most common soft tissue sarcoma in childhood; it is located mainly in the head and neck, followed by the genitourinary tract and the limbs.^3^^,^^13^^,^^14^

There are other soft tissue sarcomas in adults, such as liposarcomas, histiocytomas, angiosarcomas, and leiomiosarcomas.^13^

These tumors have a bimodal peak incidence, the first peak occurring in children aged from 2 to 6 years and the second peak occurring in adolescence. There is a slight male predisposition for sarcomas tumor.^13^^,^^14^

The mean age in out sample was 7.79 years; males predominated slightly (54.17%) compared to females (45.83%). Lyos et al. found similar results in a study of 134 patients where the mean age was 7 years and the male to female ratio was 1.2:1.^14^

Quaglia et al. also found a bimodal age distribution with a peak in the first two years of life and another in late adolescence. In their study, the age at diagnosis had an independent effect on the survival of patients with invasive, but not metastatic, tumors. The few reports of rhabdomyosarcomas in adult have sown that survival is worse compared to children, notwithstanding multimodal therapy.^15^

The embryonic subtype was the most common in our review, followed by the alveolar subtype; according to the literature, these are also the most common forms in children. In three children with parameningeal alveolar rhabdomyosarcomas, two had distance metastases (one in the meninges and one in bones and lungs) and died. The third patient have local invasion to the temporal bone, and responded to therapy; this patient was disease-free in the follow-up period. Although botryoid tumors have a better prognosis according to published results, our patient with this subtype had local invasion to the posterior fossa and metastases to bones, meninges and the central nervous system. We found no follow-up data on this patient. No pleomorphic subtype was found, which concurs with published results that suggest this tumor occurs almost exclusively in adults.^10^

Parameningeal sites comprise half of all cases of head and neck rhabdomyosarcomas and 17% of rhabdomyosarcoma cases in general. The IRS-I considers the head and neck as unfavorable sites because critical anatomical structures may be invaded, complete resection may not be possible, and early leptomeningeal invasion may occur. Furthermore, these sites are less visible compared to superficial head and neck sites, which implies in delayed diagnoses.^7^^,^^16^ The orbit as a site comprises about 25 to 30% of head and neck rhabdomyosarcomas, and 10 to 20% of all rhabdomyosarcomas;^11^ the prognosis is better compared to tumors in parameningeal sites.^12^^,^^17^ The three patients with orbit rhabdomyosarcomas had no intracranial invasion or metastases at the time of diagnosis. The two patients with non-parameningeal tumors had no intracranial invasion, but had distance metastases. Of 19 patients with parameningeal tumors, three had no intracranial invasion or metastases, which concurs with the literature that this site has a worse prognosis.

Neves et al. found that 100% of patients with rhabdomyosarcomas in parameningeal sites died mostly because of tumor progression, confirming that tumors in this site have a worse prognosis. In this study, the orbit was the most frequent site in head and neck; it had the lowest death rate, which was mostly due to complications of therapy.^13^

The clinical response to treatment depends on the site of the primary tumor, the histological subtype, the tumor size, the patient's age, and the extent of the disease.^3^, ^5^, ^18^, ^19^, ^20^

International study protocols such as those developed by the United States rhabdomyosarcoma intergroup^5^ in 1972, and the malignant mesenchymal tumor study (MMT) which the International Society of Pediatric Oncology (SIOP) undertook in Europe, have increased our knowledge about this disease and improved the survival of patients with rhabdomyosarcomas.^5^^,^^17^ The long-term survival rate for these patients with non-metastatic disease is above 70%.^20^^,^^21^ However, tumors are generally diagnosed when they are at an advanced stage with local invasion.^8^

Authors in the literature have advocated multimodal therapy for rhabdomyosarcomas; this involves individualized systemic chemotherapy, surgery, and radiotherapy, according to the initial presentation of the tumor.^2^^,^^5^^,^^21^ The IRS recommends surgery at first if there will be no functional and/or esthetic loss, followed by chemotherapy. Patients where the tumors are fully resected have a better prognosis. Some patients with initially non-resectable tumors may undergo surgery for removing residual tumor mass after chemotherapy. Radiotherapy is indicated for alveolar subtype rhabdomyosarcomas or in patients with residual tumors after the initial treatment.^5^^,^^10^^,^^18^ Hidden lymph node metastases are uncommon; therefore, preventing neck dissection is not indicated. Surgery is not always feasible, especially in difficult anatomical areas or for tumors that invade intracranial structures.^17^

MMT comprises chemotherapy followed by alternative chemotherapy if there is a poor response to the initial treatment; the aim is to avoid local therapy because of its complications (morbidity of surgery and the side effects of radiotherapy).^5^ The gold standard is an association of vincristine, cyclophosphamide and actinomycin-D.^5^^,^^18^

Jochen et al. carried out a retrospective study of 15 nose and paranasal sinus rhabdomyosarcoma cases, and reported that there was a trend for superior clinical responses in patients with embryonic rhabdomyosarcomas and no intracranial invasion and those patients treated with surgery followed by radiochemotherapy. Complications were seen in 6 of 15 patients that underwent radiotherapy; these included epilepsy, cerebral radionecrosis, optic nerve radionecrosis, growth disorders, and polyneuropathy.^8^

Beverly et al. reviewed the data of 47 patients with superficial rhabdomyosarcomas of the face and concluded that these patients often presented resectable located lesions upon the diagnosis. Favorable prognostic factors included age below 12 years, the female sex, the embryonic subtype, and absence of lymph node metastases.^18^

Our review showed that all patients underwent chemotherapy, as defended in the literature. Alternative therapy was given to 19 patients; 15 of these patients underwent radiotherapy and 4 underwent surgery. Survival data were not available.

## CONCLUSION

Head and neck rhabdomyosarcomas often present non-specific symptoms. Consequently, delays in the diagnosis and therapy occur in many cases, especially in parameningeal tumors. Early identification optimizes the potential for cure and decreases the morbidity of therapy, again especially in parameningeal tumors.

Individualized multimodal therapy is needed for these patients; it depends on the tumor size and site, the histological subtype, local invasion, and distance metastases. Surgery should be done as the first treatment if it causes no functional or esthetic harm, followed by systemic chemotherapy. Radiotherapy is indicated for the alveolar subtype of rhabdomyosarcomas or for patients with residual tumors following the first treatment.
